# Rewiring immunity: Midkine’s emerging role in cancer immune escape and drug resistance

**DOI:** 10.1016/j.isci.2026.115497

**Published:** 2026-03-27

**Authors:** Minakshi Saikia, Sai Prem, Vaishali Kapoor

**Affiliations:** 1 Department of Radiation Oncology, Washington University in St. Louis School of Medicine, St. Louis, MO, USA; 2 Siteman Cancer Center, St. Louis, MO, USA

**Keywords:** immunology, cancer

## Abstract

Immunotherapy has transformed cancer care, yet durable responses remain limited by mechanisms of resistance driven by tumors and their microenvironment. Midkine (MDK), a secreted heparin-binding growth factor, has recently emerged as a regulator of the cancer-immune interface. While MDK has long been recognized for its roles in cell survival, proliferation, and angiogenesis, new evidence highlights its ability to reprogram the tumor microenvironment by recruiting myeloid-derived suppressor cells, impairing T cell activation, and disrupting antigen presentation. MDK further reinforces immune evasion and therapeutic resistance through crosstalk with immune checkpoints, stromal components, and signaling pathways, including STAT3, PI3K/AKT, and the Wnt/β-catenin pathway. Here, we summarize current knowledge of MDK’s immunoregulatory functions across various cancer types and propose a conceptual framework that positions MDK as a converging node linking oncogenic signaling with immune escape. We also discuss emerging translational strategies to inhibit MDK to overcome therapy resistance and enhance patient outcomes.

## Graphical abstract

**Figure:**
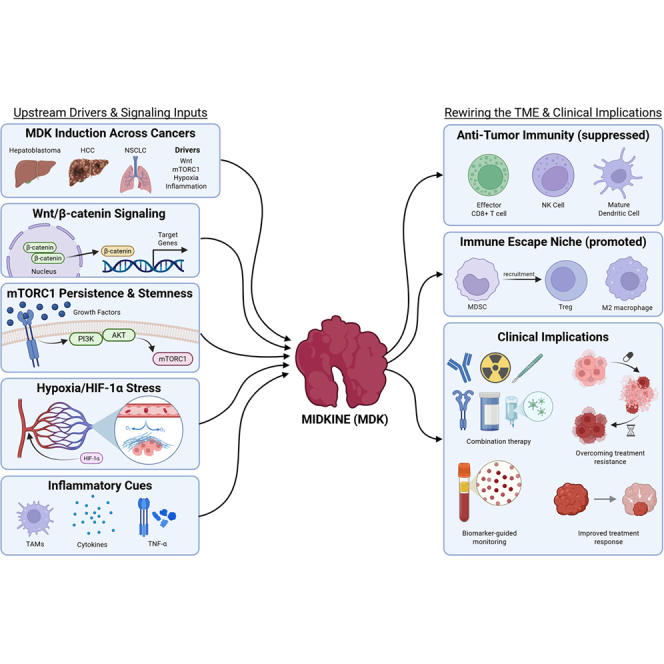

## Introduction: midkine at the intersection of cancer and immunity

The immune system comprises cells that participate in regulating the body’s defense mechanisms. It is designed to prevent and fight off any invading foreign particle, along with keeping a check on the recognizing self and non-self-cells.^1^ As such, cancer cells also develop neoantigens,^2^ which the body’s immunity is supposed to fight off. But most of the time, this smart group of cancer cells modulates the microenvironment, comprising the immune cells, in their favor for their growth and proliferation.^3^ Therefore, the immune system is a double-edged sword that can retard or support cancer propagation.^4^ Tumor immune dynamics refers to the complex interplay between the host’s immune system and tumor cells. Immunotherapy involves employing the body’s immune cells to eradicate the malignant cells^5^^,^^6^ and comprises different modalities such as immune checkpoint inhibitors (ICIs), CAR-T cell therapy, adoptive cell transfer (ACT) therapies, mRNA-based vaccines, oncolytic viruses (OV) therapy, bispecific antibodies, and monoclonal antibodies (mAbs).^7^

Immunotherapy is now one of the significant and strong pillars of cancer therapy.^4^ However, it has some limitations, and the goal of complete remission from the disease has not yet been achieved. Therapies like ACT and ICIs count on immune cell activation. In contrast, tumors secrete immunosuppressive molecules, such as TGF-β and IL-10, and recruit T-regulatory cells (Tregs) and myeloid-derived suppressor cells (MDSCs) within the tumor microenvironment (TME), which dampen the therapeutic effects of immune cells. Tumor heterogeneity, T cell exhaustion, tumor mutational burden (TMB), and other immune evasion mechanisms pose a detrimental impact on the success of immunotherapy.^7^

Midkine (MDK) is a secreted, heparin-binding growth factor with documented immunoregulatory activity in cancer and inflammatory settings.^8^^,^^9^ MDK is closely related to pleiotrophin (PTN), and the two proteins comprise a small family with about 50% sequence identity.^10^^,^^11^ Both ligands can engage overlapping receptor systems, including PTPRZ1 and ALK, supporting at least partial receptor-level redundancy.^12^^,^^13^^,^^14^ Consistent with compensatory biology, PTN transcription increased in an organ-specific manner in MDK-null mice, and combined MDK and PTN deficiency produced more severe developmental phenotypes than either single knockout.^11^^,^^15^^,^^16^ In human gliomas, MDK and PTN co-expression associated with poorer outcomes than either factor alone, suggesting that both ligands may be active in the same tumor context.^17^ However, tumor studies also show tumor-associated macrophages (TAMs) can secrete PTN to stimulate PTPRZ1 signaling in glioblastoma stem cells, linking PTN to myeloid-rich niches.^18^ In metastatic breast cancer models, PTN promoted a pro-metastatic immune microenvironment, and PTN blockade enhanced the activity of immune checkpoint blockade and chemotherapy, indicating that PTN can contribute to immune escape and therapy resistance.^19^ By contrast, melanoma-secreted MDK impaired dendritic cell (DC) differentiation and function and limited response to immune checkpoint blockade, supporting a direct role for MDK in immunotherapy resistance.^9^^,^^20^ Together, these findings motivate evaluating PTN as a potential parallel or compensatory axis when developing MDK-directed strategies, particularly in tumors with MDK and PTN co-expression or strong myeloid infiltration.^15^^,^^17^^,^^18^

MDK levels are elevated in different cancers, such as bladder,^21^ breast,^22^ pancreatic ductal adenocarcinoma (PDAC),^23^ soft tissue sarcoma,^24^ melanoma,^25^ hepatocellular carcinoma (HCC),^26^ lung,^27^ brain,^17^ esophagus,^28^^,^^29^ and ovary.^30^ Since MDK is a secretory cytokine, researchers have demonstrated that MDK can be exploited as a non-invasive serum biomarker for different cancer types.^31^^,^^32^^,^^33^^,^^34^^,^^35^ Additionally, MDK can be a promising therapeutic target for cancer. iMDK, a small molecule inhibitor of MDK expression, has been shown to retard tumor cell growth in preclinical studies. iMDK is effective either alone or in combination with other therapeutics^36^ in minimizing tumor burden and increasing the overall survival of mice bearing cancers such as lung,^37^^,^^38^ oral squamous cell carcinoma (OSCC),^39^ multiple myeloma,^40^ and HCC.^41^ A new inhibitor of MDK, namely HBS-101, has been developed, which binds to its endogenous receptor binding site, thereby impairing its biological functions. This inhibitor is reported to have promising results in triple-negative breast cancer (TNBC) therapy.^42^

MDK also functions as a proinflammatory cytokine, contributing to chronic inflammation by facilitating the chemotaxis and infiltration of neutrophils and macrophages into tissues.^43^ In addition to its well-established roles in cell growth, proliferation, and angiogenesis,^44^ this inflammatory activity contributes to the creation of a tumor-supportive microenvironment, enabling cancer cells to survive, adapt, and thrive.^45^^,^^46^^,^^47^^,^^48^ MDK has been reported to reprogram the body’s immune cells, such as MDSCs and macrophages, thereby creating an immunosuppressive environment within the tumor. This results in therapy resistance and tumor immune evasion.^9^^,^^47^

The objective of this review is to provide a comprehensive evaluation of MDK’s role in cancer immune evasion and drug resistance. We will examine and dissect the diverse molecular pathways through which MDK facilitates immune escape and contributes to therapeutic resistance. By integrating current findings, this review aims to establish a framework for understanding the therapeutic potential of targeting MDK, elucidating resistance mechanisms, and identifying emerging clinical opportunities.

## MDK biology and mechanisms of immune modulation

### MDK structure, signaling, and regulation in cancer

**Figure 1:**
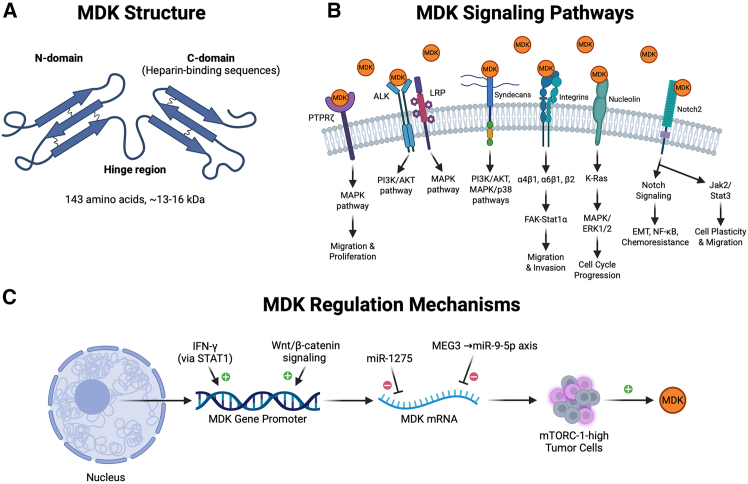
Structural architecture, multi-receptor signaling, and regulatory landscape of MDK (A) MDK protein structure highlighting the 143 amino acid sequence (∼13–16 kDa) composed of an N-domain and a C-domain connected by a hinge region, with each domain containing three anti-parallel β-strands and stabilized by a total of five disulfide bonds. The cysteine-rich C-terminal domain serves as the primary site for heparin-binding and functional protein interactions. (B) MDK signaling network showing the interaction with diverse receptors including PTPRZ, ALK, and LRP to activate MAPK and PI3K pathways for proliferation; syndecans to regulate AKT and p38 signaling; integrins to induce FAK-Stat1α-mediated invasion; nucleolin to drive K-Ras/Erk cell cycle progression; and Notch2 to facilitate EMT, NF-κB overexpression, and Jak2/Stat3-mediated cell plasticity. (C) Multilevel regulation of MDK expression, featuring transcriptional induction by IFN-γ/STAT1 and Wnt/β-catenin signaling, post-transcriptional repression by miR-1275 and the MEG3 → miR-9-5p axis, and metabolic maintenance by mTORC1-high signaling in stem-like cellular states to foster immune suppression. Created in https://BioRender.com.

MDK is a small protein composed of 143 amino acids and has a molecular weight of approximately 13–16 kDa. MDK is also referred to by its gene aliases MK1 and NEGF2 (neurite growth-promoting factor 2).^49^^,^^50^ It has two distinct domains, the N-domain and the C-domain, connected by a hinge region, and each domain consists of three anti-parallel β-strands (Figure 1A). Three disulfide bonds stabilize the N-domain, and two disulfide bonds stabilize the C-terminal.^51^ The cysteine-rich C-terminal domain contains the heparin-binding sequences and interacts with other proteins to carry out the different biological functions^52^ (Figure 1A).

MDK can bind to various receptors, including protein tyrosine phosphatase zeta (PTPRZ),^53^ anaplastic lymphoma kinase (ALK),^54^ low-density lipoprotein receptor-related protein (LRP),^55^ syndecans, integrin, nucleolin, and Notch2, to initiate a plethora of downstream processes that regulate oncogenesis^54^^,^^56^ (Figure 1B). The Arg78 amino acid in MDK at its C-terminal domain plays an essential role in high-affinity binding to cell surface moieties on PTPRZ.^57^ The binding of MDK to PTPRZ, a chondroitin sulfate proteoglycan, activates the mitogen-activated protein kinase (MAPK) pathway, facilitating migration and proliferation of cancer cells^58^ along with the phosphorylation of Akt and Syk and elevation of Bcl2 levels.^59^ Reports also indicate this initiates a signaling cascade leading to B cell survival via the activation of the MIF/CD74 pathway.^53^ Interaction of MDK with the ALK and LRP receptors subsequently activates the phosphatidylinositol 3-kinase (PI3K) and MAPK pathways.^60^^,^^61^ The binding of MDK with another proteoglycan, syndecan (syndecan 1/3/4), has been found to regulate the PI3K/AKT, MAPK/p38 signaling pathways.^56^^,^^62^ α4β1 integrins and α6β1 integrins form an MDK receptor complex.^8^ MDK is reported to induce migration and invasion in head and neck squamous cell carcinoma (HNSCC) via the FAK-Stat1α signaling pathway by promoting tetraspanin-integrin interaction.^63^ It has been referred to as a middle manager of β2 integrins.^64^ MDK interacts with nucleolin^65^^,^^66^ and activates K-Ras and subsequently the MAPK/Erk1/2/cyclin D1 signaling that results in enhanced cell cycle progression.^67^ MDK is identified as an anti-HIV protein due to its binding with the cell surface nucleolin receptor.^68^ Knock down of nucleolin using siRNA suppressed MDK-induced EGFR activation *in vitro*, and a chemical inhibitor of nucleolin, AS1411, suppressed proliferation, and migration of pulmonary arterial smooth muscle cells (PASMC) induced by MDK.^65^ Interaction of MDK with Notch2 activates the Notch signaling, resulting in upregulation of epithelial-to-mesenchymal transition (EMT), overexpression of NF-κB, and development of chemoresistance.^69^ Reports also indicate that cross-talk between Notch2/Jak2/Stat3 regulates cell plasticity and migration.^47^^,^^54^^,^^70^

Interferon gamma induces MDK via STAT1 across models, with cervical and ovarian systems showing dose-dependent induction and reversal of pro-metastatic effects when MDK is blocked^71^^,^^72^^,^^73^ (Figure 1C). Wnt/β-catenin signaling also upregulates MDK, exemplified in non-small cell lung carcinoma (NSCLC), where antibody blockade of the TIP1 functional domain activates β-catenin and increases MDK, an effect abolished by β-catenin silencing.^36^ Post-transcriptional control contributes as well; miR-1275 directly targets the MDK 3′UTR in breast cancer and associates with chemoresistance, while a MEG3 → miR-9-5p axis restrains MDK in HCC.^74^^,^^75^ Finally, MDK links to cellular state and nutrient signaling, since stem-like, mTORC1-high tumor cells sustain MDK to persist under rapalog pressure and foster an immune-suppressive niche.^45^ MDK’s structure, its binding to different receptors to activate corresponding signaling pathways, and the mechanisms regulating MDK expression are illustrated in Figure 1.

### MDK’s role in shaping an immunosuppressive TME: recruitment of MDSCs, suppression of T cells, and impact on antigen presentation

**Figure 2:**
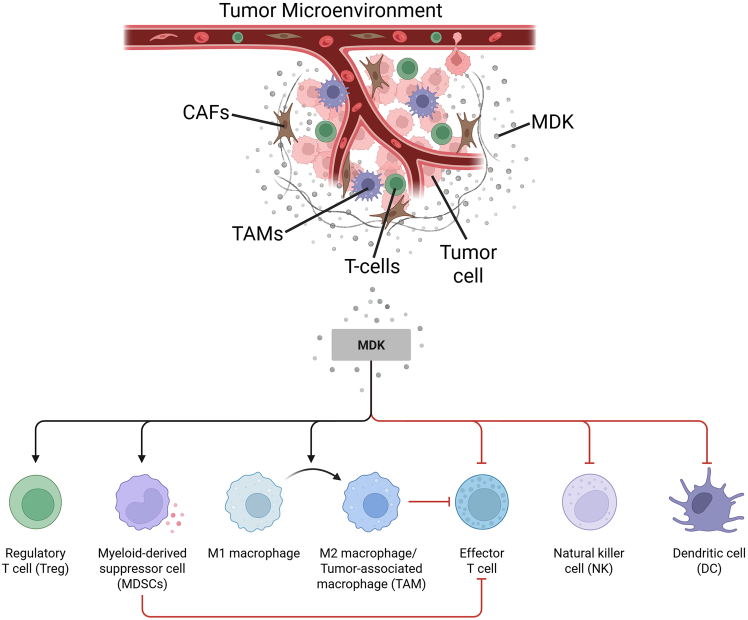
Overview of MDK-mediated immune suppression mechanisms A vascularized TME with cancer cells, CAFs, and infiltrating immune cells is shown. Soluble MDK (gray dots) accumulates locally and (1) promotes regulatory T cells and MDSCs, (2) polarizes macrophages from an M1 state to M2 or TAMs, (3) inhibits effector CD8 T cells and natural killer cells, and (4) impairs DC activation and antigen presentation. Black arrows denote promotion or polarization; red T bars denote inhibition. The net effect is an immune-cold niche with reduced antitumor activity. Created in https://BioRender.com.

Recently, MDK has been increasingly recognized as an immunomodulator that promotes tumor growth and sustenance. The key players that regulate our immune system are the cytotoxic T lymphocytes, B cells, antigen-presenting cells (APCs), and natural killer (NK) cells. MDK shapes an immunosuppressive microenvironment by adopting different strategies, such as recruiting MDSCs,^47^ suppressing T cell functions,^9^^,^^20^ and interfering with antigen presentation.^20^^,^^76^ Figure 2 shows an overview of the mechanisms by which MDK mediates immunosuppression.

MDSCs are a group of immature myeloid cells that exhibit immunosuppressive functions, which can contribute to tumor growth.^77^ MDSCs mediate immunosuppression^78^ by induction of M2 macrophages or regulatory T cells,^79^ impairment of lymphocyte adhesion onto endothelial cells, free radical production such as reactive oxygen (ROS) and inducible nitric oxide synthase (iNOS)^80^ leading to increased cyclooxygenase 2 (Cox-2), hypoxia-inducible factor 1-alpha (HIF-1α) and arginase 1 (ARG1) expression and reduced T cell receptor (TCR) expression.^81^ In HCC, the overexpression of MDK promotes the expansion and infiltration of MDSCs, thereby fostering an immunosuppressive TME and contributing to resistance against Sorafenib, the standard treatment for advanced HCC.^47^^,^^82^

Regulatory T cells (Tregs) are programmed to distinguish between self and non-self and prevent autoimmune diseases; however, cancer cells utilize these cells to create an immunosuppressive environment. Syndecan 4 (SDC4) is a receptor for MDK and is associated with Tregs, facilitating their infiltration and enhancing their motility within the TME.^46^ MDK is reported to recruit and modulate the myeloid cells toward suppressive phenotypes, leading to the polarization of the TAM toward an immune suppressive state that inhibits CD8^+^ T cell cytotoxicity.^9^^,^^83^ Recent spatial and single-cell transcriptomic analyses further demonstrated that stem-like tumor cells with high MDK expression recruit TREM2^+^/TYROBP^+^ macrophages, which contribute to T cell dysfunction and immune evasion.^45^

Antigen presentation is an essential step for the initiation of adaptive immunity, where DCs, macrophages, and B cells present the antigenic peptides on their surface bound to the major histocompatibility complex (MHC) for activation of the T cells. MDK has been shown to activate STAT3 signaling, impairing the differentiation, activation, and function of DCs, particularly conventional type 1 DCs (cDC1s). This disruption leads to defective antigen presentation and diminished priming of CD8^+^ T cells.^20^ MDK suppresses the development of tolerogenic DCs (DCregs). This drives the development of Tregs, which dampens immunity in the TME.^84^ Further, reports have indicated that MDK reprograms the macrophages and recruits MDSCs that directly interfere with antigen presentation.^9^ Collectively, these findings position MDK as one of the central regulators of immune suppression within TME.

### Crosstalk with immune checkpoints and stromal components

Engaging the immune checkpoints and stromal components simultaneously helps MDK orchestrate immune evasion. A study demonstrated that simultaneous targeting of immune checkpoints and MDK in melanoma results in synergistic antitumor efficacy.^9^ Another study showed that in HCC, MDK enhances MDSC infiltration into the TME, followed by IL-10 production via the NF-κB/STAT3 pathways, thereby dampening CD8^+^ T cell infiltration and attenuating the efficacy of PD-1 blockade. Whereas blocking MDK could reverse the effectiveness of anti-PD-1 immunotherapy.^47^ Stromal elements such as cancer-associated fibroblasts (CAFs) have also been reported to secrete MDK. This CAF-derived MDK contributes to tumor cell survival and enhances tumor resistance to chemotherapy, such as cisplatin.^85^ Mechanistically, CAF-derived MDK programs chemoresistance in multiple tumors. In gastric cancer, CAF-secreted MDK upregulates the lncRNA ST7-AS1 in tumor cells, activating PI3K/AKT signaling and EMT programs that raise the cisplatin IC50 and blunt apoptosis; neutralizing MDK or silencing ST7-AS1 restores chemosensitivity and reverses resistance in SGC-7901/DDP xenografts.^86^ In OSCC, CAF-secreted MDK induces the lncRNA ANRIL to promote cisplatin resistance, and blockade of MDK or ANRIL knockdown re-sensitizes tumor cells.^85^

### Connections to known cancer cell signaling pathways

A review of the literature can establish MDK as a hub of the major cell death inhibitory pathways, which help cancer cells thrive within the body. Binding MDK to receptors such as Notch2, LRP1, or transforming growth factor β (TGF-β) activates the Janus Kinase (JAK)/signal transducer and activator of transcription (STAT) as well as MAPK pathways.^87^ In HCC, an insulin-like growth factor 1 receptor (IGF-1R) promoted Stat3 activation, which in turn upregulated MDK, and a Stat3-MDK-Stat3 positive feedback loop was observed.^88^ Glioblastoma progression is reported to be promoted by MDK via the PI3K/Akt signaling pathway.^89^ MDK-induced MDSCs have been found to activate NF-κB, Akt, ERK, and STAT3, along with enhanced production of IL-10, which contributes to T cell suppression.^47^ WNT-β-catenin signaling can be considered as one of the prime signaling pathways regulating oncogenesis. Reports have shown that MDK expression is regulated by the WNT-β-catenin signaling in glioma^50^ and NSCLC.^36^ Oncogenic WNT-β-catenin signaling blunts T cell activation and recruitment.^90^ These signaling pathways, upregulated by MDK within the tumor, aid the cancer cells in gaining survival, stemness, and immune evasion properties.

## Context-specific roles of MDK in tumor progression and resistance

MDK functions vary with tumor lineage, stage, and microenvironmental context, shaping disease behavior and therapeutic response. Representative tumor-specific evidence is consolidated in Table 1, and the subsections below synthesize context-specific differences and mechanisms, which highlight potential points for therapeutic intervention.

**Table 1:** Comparative role of MDK across cancer types

Cancer type	Evidence MDK is upregulated/active	Dominant receptors or pathways implicated	Immune-context effects most often reported	Clinical associations/resistance	Therapeutic implications	Reference
Hepatocellular carcinoma (HCC)	Sorafenib increases intratumoral MDK; MDK drives myeloid skewing	MDK to MDSC IL-10 up; PI3K to AKT and JAK to STAT activation in myeloid cells	Increased MDSC infiltration; increased PD-L1 and IL-10; reduced CD8 T cell function	Anti PD-1 efficacy blunted in sorafenib treated, MDK high settings	MDK inhibition restores anti-PD-1 activity and reduces MDSC suppressive function	Okada et al.^34^; Ding et at.^47^
Melanoma	Tumor secreted MDK broadly elevated in aggressive disease	STAT3 mediated reprogramming of DCs, especially cDC1	DC differentiation and activation impaired; reduced cross-priming; systemic APC dysfunction	MDK high DC signature associates with poor prognosis and ICB resistance	MDK suppression enhances DC-targeted vaccination, CD40 agonism, and ICB responses	Catena et al.^20^
Colorectal cancer (CRC)	Spatial and single-cell analyses identify MDK high tumor regions	MDK engages SDC4 on Treg cells	Co-localization of MDK with Treg infiltration; immunosuppressive niches	Suggests MDK-linked immune exclusion in early carcinogenesis	MDK blockade proposed to limit Treg recruitment and retention	Hashimoto et al.^46^
Glioblastoma (GBM)	MDK overexpressed vs. normal brain; correlates with worse survival	MDK to ALK to PI3K to AKT to ERK to STAT3; MDK driven GIC maintenance	Stem-like programs sustained; indirect immune dampening via tumor intrinsic signaling	Higher MDK links to poorer outcomes	ALK or MDK targeting reduces GIC self-renewal; sensitizes to temozolomide	Hu et al.^89^; López-Valero et al.^91^
Non-small cell lung cancer (NSCLC)	Hypoxia and HIF-1a upregulate MDK; MDK promotes angiogenesis and metastasis	HIF-1a to MDK; MDK to Notch2 or NF-kB; iMDK active in models	Pro-angiogenic, pro-metastatic microenvironment; potential myeloid skewing	Elevated tissue or serum MDK associates with worse survival; biomarker potential	MDK inhibitor iMDK limits growth and metastasis *in vivo*	Xia et al.^32^; Shin et al.^38^
Hepatoblastoma (pediatric)	Multiomic data show WNT responsive MDK in tumor cells	WNT or beta catenin to MDK; macrophage phenotype modulation	Increased anti-inflammatory macrophage states; immune exclusion signatures	MDK linked to immune evasion and poor differentiation programs	MDK inhibition partially reverses macrophage reprogramming *ex vivo*	Munter et al.^92^
TSC associated tumors (mTORC1 driven)	MDK enriched in mTORC1 hyperactive or TSC contexts	MDK sustains stem like, drug persistent states under mTOR inhibition	Immunosuppressive TME programs with MDK high tumor cells	Persistence to rapalog therapy linked to MDK high states	Combine MDK blockade with mTOR inhibitors to reduce persistence and remodel TME	Tang et al.^45^
Pancreatic ductal adenocarcinoma (PDAC)	Elevated MDK in tumors or serum and cancer cell EVs; MDK supports proliferation and migration	LRP1, SDC1 or 4, ALK; downstream MAPK or PI3K signaling; inflammatory inducers such as TNF-a or EGF	MDK-targeted nanobody PDT remodels the TME toward improved antitumor immunity in preclinical models	High MDK aligns with aggressive biology; candidate biomarker	Antibody or nanobody based MDK targeting; potential synergy with ICI after microenvironment reprogramming	Rawnaq et al.^23^; Qu et al.^93^
Gastric cancer	Tumor or serum MDK is increased; MDK drives malignant phenotypes	p38 MAPK to CHOP or AP-1 axis linked to MDK induced stress ligand expression	MDK upregulates MICA or B and reduces NK cytotoxicity, fostering immune evasion	Elevated MDK associates with progression	MDK inhibition and/or NK restorative strategies such as MICA or B directed approaches	Zhao et al.^94^
Gallbladder cancer	Single cell atlas shows MDK dependent immunosuppressive ecosystem in ErbB mutant tumors	Context-dependent MDK signaling such as NCL or LRP1, crosstalk with ErbB driven programs	Enrichment of suppressive myeloid populations and dysfunctional T cells in MDK high niches	MDK high state may underlie poor immunotherapy responsiveness	MDK blockade to de suppress TME; combine with checkpoint blockade	Zhang et al^95^
Clear cell renal cell carcinoma (ccRCC)	MDK expression functionally promotes M2 macrophage polarization	LRP1 or NCL and downstream STAT3 or AKT context dependent	TAM skewing toward M2 state; T cell exclusion or dysfunction signatures	MDK associates with adverse features and immune cold states	MDK inhibition to re-educate TAMs and potentiate PD-1 or PD-L1 therapy	Shi et al.^96^
Endometrial carcinoma	Spatial and single cell profiling identifies an MDK to NCL dependent immunosuppressive niche	MDK to nucleolin axis	Reduced antitumor immune activity within MDK high regions	Potential for MDK status to stratify immunotherapy benefit	Target MDK to NCL axis; rational ICI combinations	Yu et al.^48^
Breast cancer, inflammatory subtype	CD151 to MDK pathway controls immune composition via EV associated MDK	CD151 to alpha6beta1 to MDK signaling; EV-mediated cytokine or chemokine programs	Monocyte or macrophage recruitment; pro-tumor macrophage polarization	Links between MDK axis, macrophage infiltration, and treatment response	Blockade of MDK or CD151 axis; TAM reprogramming strategies	Hayward et al.^97^
Prostate cancer (CRPC or NE differentiated)	MDK associates with neuroendocrine differentiation; MDK inhibition reduces stem-like populations	NF-kB inducible MDK; NE lineage programs	Immune excluded, cytokine rich niches typical of NE CRPC; MDK may reinforce non-inflamed states	NE differentiation linked to AR-targeted therapy resistance; MDK marks aggressive disease	Small molecule or biologic MDK inhibitors; combinations with AR pathway or ICI in selected contexts	Erdogan et al.^98^; Nordin et al.^99^
Bladder cancer	MDK overexpressed in tumors; elevated urinary MDK as a noninvasive marker; MDK links to poor outcome in invasive disease	Angiogenic MDK signaling; canonical receptors such as LRP1 or SDCs	Urothelial TME often immunosuppressive; MDK aligns with aggressive phenotypes	Prognostic and diagnostic utility using urine testing	MDK as surveillance biomarker; exploratory targeting in high-risk disease	O’Brien et al.^21^; Lin et al.^100^
Papillary thyroid carcinoma	Serum MDK tracks disease status after therapy; tumor overexpression reported	Not fully defined; MDK-linked survival and angiogenesis pathways likely	Immune context variable; MDK primarily a biomarker signal here	Dynamic MDK changes correlate with metastatic risk and monitoring	MDK as adjunct disease monitoring marker; exploratory targeting	Li et al.^101^
Hematologic malignancy	qPCR analysis revealed higher MDK levels	Clinical trial	Clinical trial	Higher MDK links to poorer childhood leukemia outcomes	Prognostic marker and a tool for disease monitoring in childhood ALL	Hidaka et al.^102^; Jia et al.^103^

### Hepatocellular carcinoma: Sorafenib resistance, MDSC infiltration, synergy with PD-1 blockade

Sorafenib, a multi-kinase inhibitor with antiangiogenic properties, is an FDA-approved first-line treatment option for advanced HCC.^104^ Prolonged exposure to sorafenib leads to intratumoral hypoxia and induces MDK expression, along with PD-L1 and other immunosuppressive cytokines, such as IL-10 and TGF-β. MDK mediates the accumulation and activation of MDSCs and suppresses the CD8^+^ T cell proliferation and activity. PD-1 blockade was found to be largely ineffective in Sorafenib-mediated chemotherapy, but PD-1 blockade, along with MDK inhibition showed beneficial effects in Sorafenib-treated tumor reduction via restoration of effector T cells in the tumor.^47^ Younis et al. have demonstrated using both *in vitro*^105^ and *in vivo*^106^ studies that codelivery of lipid nanoparticle containing Sorafenib and siRNA against MDK in HCC is a highly promising strategy for HCC therapy.

### Melanoma and colorectal cancer: MDK’s impact on T cell infiltration and immune evasion via Wnt signaling

In melanoma, MDK has been demonstrated to create an inflamed but immune-refractory TME. MDK facilitates the activation of NF-κB and secretion of proinflammatory cytokines. This helps in the recruitment of MDSCs and macrophages (hence, inflamed). Still, at the same time, MDK represses type I interferon and IFN-γ-related signaling, which are critical for T cell recruitment and priming (creating an immune refractory environment). Under these circumstances, the macrophages are modified toward a tolerogenic phenotype, which prevents the activation of CD8^+^ cytotoxic T cells, leading to immune evasion.^9^ MDK also impairs the antigen presentation and T cell priming via activation of the STAT3 pathway, thereby mediating DC dysfunction and resulting in immune suppression.^20^ MDK secreted by tumor cells in colorectal cancer has been shown to interact with syndecan-4 (SDC4) on regulatory T cells (Tregs) and promote their infiltration and motility, contributing toward an immunosuppressive microenvironment that hinders effective CD8^+^ T cell responses.^56^

### Immunosuppressive myeloid niches in clear cell renal cell carcinoma, endometrial carcinoma, and gallbladder cancer

Across these tumors, a higher MDK is associated with an immune-suppressed microenvironment characterized by increased myeloid cells and reduced effective T cells. In clear cell renal cell carcinoma, MDK marks an immune-excluded state with M2-like macrophage features and worse outcomes, including weaker benefit from immunotherapy.^96^ Endometrial carcinoma shows an MDK-nucleolin signal that connects tumor cells with endothelial and immune programs that limit local antitumor activity.^48^ In gallbladder cancer, especially in ERBB-mutant disease, MDK associates with macrophage reprogramming and regulatory T cell activity, suggesting that MDK targeting could help recondition the microenvironment.^95^

### NSCLC: hypoxia, angiogenesis, and circulating MDK

In NSCLC, hypoxic stress increases MDK and links it to vascular support and spread. Elevated MDK aligns with endothelial activation, tube formation, and higher microvessel density, which sustains tumor perfusion and growth.^38^ Tumor-derived MDK also engages survival and motility pathways that favor invasion and metastasis, while small-molecule inhibition reduces primary growth and metastatic burden *in vivo*, indicating that MDK is a functional driver in this setting.^32^ Circulating MDK has been explored as a fluid readout that correlates with aggressive tumor features and could complement imaging during the assessment of tumors.^38^

### Invasion and innate immune evasion driven by MDK in PDAC and gastric cancer

In PDAC, MDK increases proliferation, motility, and invasion through MAPK or PI3K pathway activation, in line with EMT patterns and aggressive behavior; MDK-targeted, nanobody-engineered phototherapeutic approaches remodel the TME and enhance antitumor immunity *in vivo*.^23^^,^^93^ In gastric cancer, MDK upregulates the stress ligands MICA and MICB and reduces natural killer cell cytotoxicity, indicating an innate immune-evasion route that may be addressable with MDK-directed strategies.^94^

### Hepatoblastoma: MDK and differentiation states; Wnt-MDK axis in pediatric cancer

Hepatoblastoma is the most prevalent pediatric cancer of the liver, affecting children usually below 4 to 5 years of age. This cancer subtype is always associated with WNT-activating CTNNB1 mutation and is characterized by a heterogeneously differentiated population of hepatocytes.^107^^,^^108^ A reduced degradation of β-catenin due to the activated CTNNB1 mutation results in activation of other aberrant molecules such as MDK.^36^ Hepatoblastoma exhibits distinct epithelial programs that track hepatic differentiation, with fetal-like HNF4A^+^ cells contrasted by WNT-high embryonal LEF1^+^ cells and corresponding differences in drug sensitivities, including broad organoid sensitivity to Histone Deacetylase (HDAC) inhibitors and subtype-biased responses to Epidermal Growth Factor Receptor (EGFR) or Fibroblast Growth Factor Receptor (FGFR) blockade.^108^ Spatial and histology-guided transcriptomics further demonstrate that embryonal regions co-express Wnt targets with the biliary factor SOX4 and display focal FGF19 expression, which functions as a paracrine growth signal required for subsets of tumoroid cells, linking lineage state to Wnt-cooperating mitogen cues.^109^ Single-cell profiling identifies five tumor signatures and a myeloid-skewed microenvironment in hepatoblastoma, including MARCO-low TAMs that distinguish the tumor from the adjacent liver and suggest specialized macrophage-tumor crosstalk.^110^ Multiomic mapping across 15 single-cell and 22 spatial datasets demonstrates that Wnt signaling upregulates MDK in hepatoblastoma cells, shifts the macrophage phenotype toward an immune-evasive state, and increases immune exclusion in tumor regions. Furthermore, MDK inhibition partially reverses these macrophage changes.^92^ Beyond hepatoblastoma, single-cell analyses in pediatric tumors reveal a LEF1-driven Wnt embryonic program that is also present in medulloblastoma, situating MDK regulation within a Wnt-centered developmental axis relevant to childhood cancers.^111^ MDK has been reported to create an anti-inflammatory environment and support myeloid-driven immune evasion. A pro-fibrotic shift in macrophages is observed, which is reversed when MDK signaling is inhibited in the tumors.^92^

### Prostate cancer and inflammatory breast cancer: plasticity, stemness, and macrophage remodeling

In prostate cancer, MDK tracks with lineage plasticity and neuroendocrine features in advanced disease and supports CD133-positive stem-like cells and migration; inhibition of MDK reduces these behaviors in preclinical studies.^98^^,^^99^ These links place MDK within a broader set of programs that allow cells to detach from androgen dependence and adopt alternative survival routes. In inflammatory breast cancer, a CD151-integrin-MDK axis draws in monocytes and enriches macrophages, creating a tumor-supportive niche that could be remodeled by targeting MDK alongside systemic therapy.^97^ Together, these observations connect MDK with both cell-intrinsic plasticity and myeloid-driven stromal change, which may influence how tumors respond to standard treatments.

### Tuberous sclerosis complex-associated tumors: potential links via mTOR and immune suppression

Tuberous sclerosis complex (TSC) is an autosomal dominant disorder caused by the loss-of-function mutations in one of the two genes, TSC1 (hamartin) or TSC2 (tuberin). TSC tumors such as subependymal giant cell astrocytoma and renal angiomyolipoma arise from TSC1 or TSC2 loss.^112^^,^^113^ TSC can affect multiple organs in both children and adults. The clinical features of this malignancy include tumors at different sites such as the brain, skin, heart, lungs, or kidneys. It can also cause seizures and TSC-associated neuropsychiatric disorders that include autism spectrum disorder and cognitive disability.^114^ Both TSC1 and TSC2 are inhibitors of the mTOR (mechanistic target of rapamycin) signaling pathway, which is a master regulator of cell growth and proliferation; hence, a mutation in the gene leads to constitutive mTOR activation.^115^^,^^116^ Mechanistically, TSC1 and TSC2 function together as a complex that restrains mTOR signaling.^117^^,^^118^ Loss of either gene disrupts this restraint, creating an mTOR-active tumor cell state that can persist under mTOR inhibitor pressure.^45^ It was recently shown that MDK is highly expressed in the stem-like tumor cell population within TSC tumors. This observation places MDK downstream of, or tightly coupled to, the mTOR-active stem-like state that emerges after TSC1 or TSC2 loss, rather than suggesting that MDK initiates TSC pathway activation.^45^ These cells are linked to T cell dysfunction by immunomodulated macrophages, which drive an immune-suppressive environment and persistence to mTOR inhibition.^45^ In this framework, TSC1 or TSC2 loss promotes sustained mTOR signaling, which is associated with MDK-high stem-like tumor cells that shape a macrophage-driven immune-suppressive niche and contribute to resistance to mTOR inhibition.^45^

Single-cell and plasma multiomics in TSC-associated angiomyolipoma show a stromal and monocyte-macrophage enriched microenvironment, and pulmonary lymphangioleiomyomatosis features infiltrating CD206 high macrophages that are pharmacologically modifiable.^119^^,^^120^ Mechanistically relevant to MDK, cancer models demonstrate that secreted MDK sustains mTOR pathway output via maintenance of ribosomal protein S6 (RPS6) phosphorylation, and MDK-responsive cells can be tracked by this readout.^121^^,^^122^ MDK also activates PI3K-AKT signaling in glioblastoma, providing an upstream route to mTORC1, and recent literature describes MDK-mediated recruitment and reprogramming of myeloid cells with immunosuppressive cytokine circuits.^89^ These observations support a potential MDK-mTOR-myeloid link in TSC-associated tumors that could shape responses to mTOR inhibition and immune therapy.

### MDK in hematologic malignancies

Apart from solid tumors, MDK is also expressed in normal bone marrow and blood, with higher levels in CD34^+^ progenitor cells than in unfractionated marrow. In a study of 94 children with acute leukemia, MDK gene expression (normalized to K562 cells) was frequently over-expressed defined as at least twice the maximal level seen in normal bone marrow or peripheral blood—especially in B-precursor ALL (30/41 patients, 73.2%) and in more than half of French-American-British classification (FAB) M1/M2 AML cases, but was rare in T-ALL (1/13) and absent in AML-M5. MDK expression decreased with maturation along the B-cell lineage, being highest in less differentiated B-lineage leukemic blasts.^102^ A clinical trial comprising of childhood acute lymphoblastic leukemia (ALL) studied MDK mRNA and measured MDK levels in 120 children and 30 healthy controls, and patients were split into MDK(low) and MDK(high) groups at the median expression level. MDK(high) patients had higher leukocyte counts, higher peripheral blast percentages, and higher minimal residual disease levels than MDK(low) patients, and MK expression was significantly higher at relapse than at diagnosis or in sustained remission. High MDK expression was associated with worse relapse-free survival and overall survival, and multivariate analysis showed MDK(high) status to be an independent predictor of inferior overall survival.^103^

## Therapeutic implications and clinical opportunities

### MDK as a cancer biomarker

MDK is a secreted heparin-binding growth factor detectable in tumor tissue and in biofluids such as serum and urine.^32^ In hepatocellular carcinoma, elevated serum MDK is reported in a subset of patients and associates with adverse pathology and poorer survival, with concordance between circulating and tumor expression.^34^^,^^123^ In non-small cell lung cancer, studies describe diagnostic and prognostic utility for circulating MDK, with higher levels tracking aggressive biology.^31^ In bladder cancer, many reviews of urine-based assays list MDK among some of the noninvasive biomarkers with supporting clinical evidence.^100^^,^^124^^,^^125^^,^^126^ In papillary thyroid carcinoma, recent work notes MDK overexpression in tumor tissue and serum and links higher MDK to invasive behavior, supporting its role as a disease-status marker in that setting.^35^^,^^127^^,^^128^

Spatial and single-cell studies connect MDK to specific immunologic niches. In colorectal carcinogenesis, MDK colocalizes with regulatory T cell-rich regions and engages syndecan-4 on Tregs, a pattern consistent with local immunosuppression and T cell exclusion.^46^ In glioblastoma, higher tumor MDK expression correlates with shorter survival and aligns with PI3K-AKT-linked malignant programs, consistent with an immune-refractory microenvironment.^89^^,^^91^^,^^129^

### Emerging strategies to inhibit MDK: antibodies, RNAi, combination therapies

**Figure 3:**
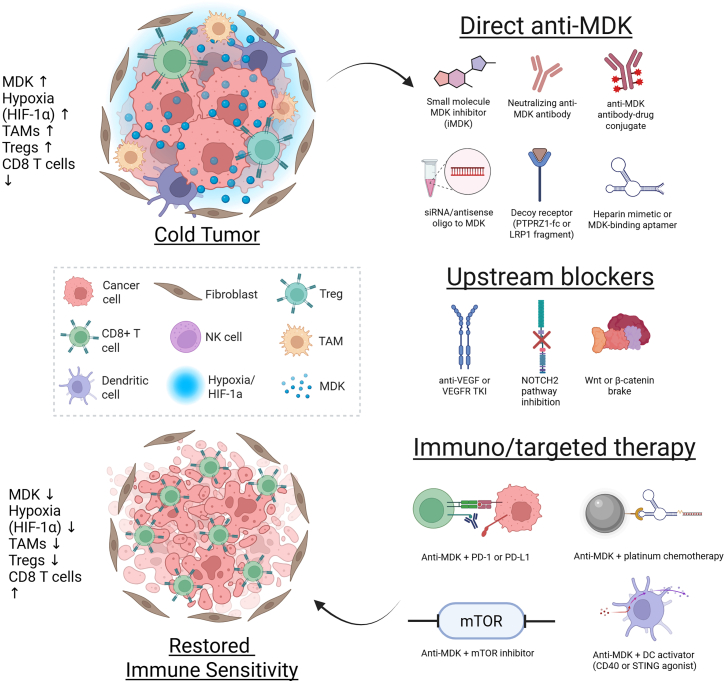
Strategies to therapeutically target MDK and restore immune sensitivity In the top left, an MDK-high, hypoxic tumor niche exhibits increased TAMs and regulatory T cells, accompanied by a sparse intratumoral CD8^+^ T cell population. The right side arranges interventions into three groups: direct anti-MDK approaches (small-molecule inhibitor iMDK, neutralizing antibody, anti-MDK antibody-drug conjugate, siRNA or antisense oligo to MDK, decoy receptor such as PTPRZ1-Fc or an LRP1 fragment, and a heparin mimetic or MDK-binding aptamer); upstream blockers (anti-VEGF or a VEGFR tyrosine kinase inhibitor, NOTCH2 pathway inhibition, and a Wnt or β-catenin brake); and combination strategies with immune or targeted agents (anti-MDK plus PD-1 or PD-L1 blockade, anti-MDK plus platinum chemotherapy, anti-MDK plus an mTOR inhibitor, and anti-MDK plus a DC activator such as a CD40 or STING agonist). The bottom left image shows the post-intervention state with MDK and hypoxia reduced, TAMs and regulatory T cells decreased or reprogrammed, and CD8 T cells increased within tissue, indicating restored immune sensitivity. Created in https://BioRender.com.

Various strategies used to target MDK are summarized in Figure 3. Ligand-directed biologics include neutralizing antibodies, engineered fragments, and payload-bearing formats. Functional anti-MDK antibodies suppressed osteosarcoma growth *in vitro* and reduced tumor burden *in vivo.*^130^^,^^131^ Antibody-drug conjugation has also been explored, with a doxorubicin-conjugated anti-MDK mAb inhibiting the growth of MDK-secreting tumor cells.^132^ More recently, MDK-targeting nanobodies have been used to deliver photodynamic therapy in PDAC, remodeling the TME and potentiating antitumor immunity *in vivo*.^93^ More recent reviews summarize antibody and nanobody modalities as future directions for MDK inhibition.^133^

Small-molecule suppression of MDK or direct ligand binding is another advancing strategy. iMDK, originally characterized as an inhibitor of endogenous MDK expression, has shown antitumor activity in multiple models, including OSCC, and has been used mechanistically to counter interferon-γ-driven metastatic programs.^39^^,^^73^ A newer, potent MDK inhibitor, HBS-101, has been developed. HBS-101 has high affinity toward MDK and interacts with three distinct residue stretches (residues 31–36, 95–100, and 107–111) by hydrophobic interactions, blocking MDK from docking to its receptors and co-receptors on tumor and stromal cells, which blocks downstream survival and motility signaling and interrupts paracrine support in the local microenvironment; HBS-101 treatment has been shown to decrease clonogenic survival and invasiveness leading to tumor reduction in TNBC xenografts, while demonstrating oral bioavailability and brain penetration in preclinical studies.^42^ The distinction between the two inhibitors is that iMDK acts upstream by suppressing MDK production inside the cell, whereas HBS-101 acts outside the cell by neutralizing secreted MDK that is already present, so HBS-101 can block signaling from both tumor and stromal sources even when transcriptional induction of MDK persists.^39^^,^^42^^,^^61^

RNA-targeting approaches silence MDK at the transcript level. In hepatocellular carcinoma, RNA interference reduced MDK expression and curtailed proliferation in a preclinical model, which supports the feasibility of gene-silencing strategies for MDK.^134^ Recent literature shows oligonucleotide therapies such as siRNA are being evaluated to lower MDK expression and adjust its signaling in cancer.^133^^,^^135^

Furthermore, combination concepts utilize MDK blockade to alleviate immune suppression or augment partner therapies. For example, in ovarian cancer models, pharmacologic or genetic MDK inhibition strengthened interferon-γ antitumor effects and reversed epithelial-mesenchymal programs.^73^ In PDAC, MDK-nanobody-guided photodynamic therapy remodeled the microenvironment and created conditions that improved immunotherapy activity in preclinical models.^93^

### Potential to overcome immune checkpoint resistance and improve targeted therapy outcomes

Multiple lines of evidence link MDK to primary and adaptive resistance to immune checkpoint blockade through effects on DCs, myeloid populations, and regulatory T cells.^20^^,^^46^ As we also described above, genetic targeting of MDK can restore immune competency and sensitize tumors to PD-1 or PD-L1 therapy in preclinical models.^20^ Independent clinical-translational studies reinforce the importance of competent cDC1 programs for anti-PD-1 responsiveness in melanoma, situating MDK’s dendritic-cell effects within a broader axis that conditions checkpoint outcomes.^136^^,^^137^ Spatial single-cell work in colorectal carcinogenesis shows MDK colocalized with regulatory T cell niches via the SDC4 axis, aligning with T cell exclusion phenotypes that are less responsive to checkpoint therapy.^46^ In parallel, immunosuppressive macrophage programs such as TREM2-positive TAM states associate with poor immunotherapy outcomes in lung cancer, consistent with the myeloid-skewing environments observed in MDK-high settings.^138^

Targeted therapy contexts provide additional links between MDK and treatment resistance. Oncogenic WNT or β-catenin activity correlates with non-T cell-inflamed states and checkpoint resistance across cancers, and MDK can be upregulated downstream of β-catenin in tumor models, suggesting an intersection between MDK induction and immune exclusion programs that undermine immunotherapy benefit.^36^^,^^50^^,^^139^^,^^140^ In clear-cell renal cell carcinoma, MDK marked an immunosuppressive subtype and predicted poor prognosis and inferior immunotherapy response, linking MDK expression to clinical resistance phenotypes in an additional solid tumor context.^96^

### Translational outlook of MDK

Because MDK is secreted and measurable in circulation, ligand-level neutralization and blood-based pharmacodynamic monitoring are feasible, at least conceptually.^20^^,^^141^^,^^142^ These features parallel TGF-β, another pleiotropic soluble pathway that promotes immune exclusion and primary resistance to PD-(L)1 blockade in preclinical models.^143^ Work in TGF-β biology also provides a template for combination development. In immune-excluded tumors, a fibroblast-centered TGF-β program correlated with poor response to PD-L1 blockade, and dual inhibition of TGF-β and PD-L1 enabled intratumoral T cell entry and tumor regression in mouse models.^144^ Consistent with this principle, engineered TGF-β×PD-L1 bispecifics such as YM101 and BiTP produced stronger antitumor activity than either monotherapy in multiple models, with immune remodeling that included enhanced DC maturation and increased CD8^+^ T cell infiltration.^145^^,^^146^ Clinically, the TGF-β trap and PD-L1 fusion protein bintrafusp alfa showed activity in early phase testing, but did not improve outcomes versus pembrolizumab in a randomized phase 3 study in PD-L1-high NSCLC, and toxicity management required careful PK and safety modeling, including bleeding risk mitigation.^147^^,^^148^^,^^149^

Similarly, MDK-directed combinations may be most compelling in tumors with myeloid-rich or immune-excluded phenotypes.^20^^,^^47^ A trial-ready starting point would be an MDK inhibitor plus PD-1 or PD-L1 blockade, with prospective evaluation of tumor and circulating MDK as enrichment biomarkers and on-treatment immune readouts such as restoration of DC programs and reductions in suppressive myeloid cells.^20^^,^^47^^,^^141^^,^^142^ Several MDK-targeting modalities have already shown *in vivo* antitumor activity, including antisense oligonucleotides,^150^ small-molecule inhibition,^39^ RNA aptamer neutralization,^151^ antibody fragment-guided payload delivery,^152^ and nanobody-engineered nanoparticles that remodel the PDAC microenvironment and can be combined with immunotherapy^93^ (Table 2). RNAi and nanomedicine strategies that pair MDK suppression with established backbones are also emerging, including combinations with sorafenib and anti-PD-1.^106^^,^^153^ Because MDK has been linked to therapy persistence under mTOR inhibition and to angiogenic programs *in vivo*, future work should test whether MDK blockade can strengthen anti-angiogenic, chemotherapy, or targeted therapy backbones while preserving safety and immune competence.^39^^,^^45^ Furthermore, if safety and early pharmacodynamic data support it, future work could explore treatment regimens that add a third target beyond MDK and PD-(L)1, such as adenosine signaling or macrophage programs.

**Table 2:** Summary of current MDK targeting strategies

Strategy class	Agent or approach	Target or mechanism	Representative context	Development stage	Reference
Direct MDK inhibitor (small molecule)	iMDK	Suppresses MDK expression and downstream signaling; used as a pharmacologic MDK inhibitor in multiple tumor models	NSCLC, OSCC, and other MDK-high tumor models	Preclinical (*in vitro* and xenograft models)	Ishida et al.^37^; Masui et al.^39^; Hao et al.^61^
Direct MDK inhibitor (small molecule)	HBS-101	Reported MDK inhibitor that blocks MDK function in MDK-driven cancers	TNBC patient-derived and brain metastasis models	Preclinical (*in vivo* efficacy reported)	Mahajan et al.^42^
Antibody-based MDK neutralization	Anti-MDK mAb	Neutralizes extracellular MDK	Osteosarcoma models	Preclinical (*in vitro* and *in vivo* proof-of-concept)	Maehare et al.^131^
Antibody drug conjugate approach	Doxorubicin-conjugated anti-MDK mAb	MDK-targeted delivery of doxorubicin to MDK-secreting tumor cells	Hepatocellular carcinoma cell model (HepG2)	Preclinical (*in vitro* proof-of-concept)	Inoh et al.^132^
Nanobody-enabled targeting	MDK nanobody-engineered nanoparticles (PDT)	MDK-targeted PDT with immunogenic cell death and microenvironment remodeling	PDAC	Preclinical (*in vivo* efficacy reported)	Qu et al.^93^
RNA interference (delivery platform)	Targeted co-delivery of sorafenib plus MDK-siRNA (lipid nanodevice)	Simultaneous kinase inhibition and MDK transcript silencing; tumor-targeted nanoparticle delivery	Hepatic cancer models	Preclinical (*in vitro* and *in vivo* efficacy reported)	Younis et al.^105^; Younis et al.^106^
Genetic MDK silencing (tool and translational proof-of-concept)	MDK knockdown (siRNA/shRNA) or pharmacologic inhibition as an immunotherapy sensitizer	Reduces immunosuppressive myeloid programs and improves response to PD-1 blockade after targeted therapy exposure	Sorafenib-treated HCC models	Preclinical (*in vivo* efficacy reported)	Ding et al.^47^
MDK receptor-axis targeting	MDK or ALK axis inhibition with temozolomide	Blocks MDK-ALK signaling to impair self-renewal and therapy resistance; combined with standard chemotherapy	Glioblastoma stem-like models	Preclinical (*in vivo* efficacy reported)	López-Valero et al.^91^
Combination therapy	iMDK plus MEK inhibitor (PD0325901)	Dual pathway suppression to enhance apoptosis and inhibit angiogenesis	NSCLC xenograft model	Preclinical (*in vivo* efficacy reported)	Ishida et al.^37^
Combination therapy	iMDK plus lenvatinib	Combines MDK suppression with multi-kinase inhibition to limit angiogenesis and myeloid polarization	HCC mouse model	Preclinical (*in vivo* efficacy reported)	Chen et al.^41^
Combination therapy	MDK inhibition plus interferon-gamma	Blocks MDK-mediated adaptive resistance to interferon-gamma and augments tumor cell apoptosis	Ovarian cancer cell line (SKOV3)	Preclinical (*in vitro*)	Liu et al.^73^
Combination therapy	MDK inhibition to counter interferon-gamma-driven EMT and metastasis programs	Pharmacologic MDK inhibition reverses interferon-gamma-STAT1-induced MDK upregulation and EMT signatures	Multiple cancer cell lines; metastasis models	Preclinical (*in vitro* and *in vivo*)	Zheng et al.^71^
Combination therapy (co-delivery)	Anti-PD-1 antibody plus MDK-siRNA co-delivery nanomedicine	Tumor-targeted delivery of checkpoint blockade with MDK transcript silencing to overcome ICB resistance	HCC orthotopic model	Preclinical (*in vivo* efficacy reported)	Xu et al.^153^

## Conclusions and future directions

MDK sits at the center of tumor-immune-stroma interactions. Across cancers, it signals through receptors such as PTPRZ, ALK, LRP1, syndecans, integrins, nucleolin, and Notch2 to drive migration, proliferation, angiogenesis, EMT, and immune escape. It reshapes myeloid biology by recruiting and reprogramming MDSCs and macrophages. MDK establishes an immunosuppressive microenvironment in the tumor by promoting M2 macrophage polarization via the MDK-LRP1 interaction, and inhibition of this cytokine suppresses M2 polarization.^96^ It impairs DC priming and tips T cell function through adenosine, ROS, iNOS, ARG1, and nutrient depletion. Its expression is tuned by hypoxia, inflammatory cues, IFN-γ-STAT1, Wnt-β-catenin, and cell state programs, with added control from copy number and miRNAs. Context matters: in HCC, MDK links hypoxia to myeloid influx and checkpoint resistance; in melanoma and colorectal cancer, it fosters inflamed yet T cell poor niches; in hepatoblastoma, Wnt activity and differentiation state track with MDK; in TSC-associated tumors, an MDK-PI3K-AKT-mTOR connection and myeloid enrichment suggest a path to combined targeting.

A key limitation of therapeutically targeting MDK is the potential for toxicity and off-target effects arising from its physiological roles in normal tissues. MDK is involved in tissue repair, angiogenesis, immune modulation, neural development, and stem cell maintenance, particularly under conditions of stress or injury.^154^^,^^155^ Systemic inhibition of MDK could therefore impair wound healing, vascular integrity, and regenerative responses, and may disrupt normal immune homeostasis.^155^ In the central nervous system, where MDK contributes to neuronal survival and plasticity, long-term blockade raises concerns about neurotoxicity or cognitive effects.^5^^,^^6^ Additionally, MDK is expressed in certain normal epithelial and endothelial tissues, increasing the risk of unintended effects on organ function. Because MDK signals through multiple receptors and pathways, including ALK, LRP1, Notch2, and integrins, its inhibition may perturb broader signaling networks, leading to unpredictable downstream consequences.

MDK has been reported to exert acute cardio-protection and neuro-protection^156^^,^^157^ against ischemia and reperfusion injury and documented to inhibit cardiac remodeling at least in part via its anti-apoptotic and angiogenic effect, respectively.^158^ HIV preventive potential of MDK is already mentioned.^68^ A latest study indicates that MDK plays a protective role against Alzheimer’s disease by preventing the accumulation of amyloid-beta, and its absence leads to increased pathology.^159^ These considerations highlight the importance of developing tumor-targeted delivery strategies, context-specific inhibitors, and biomarker-guided patient selection to minimize systemic toxicity while preserving therapeutic efficacy.

The field now needs sharper mechanistic maps that explain which MDK receptor complexes dominate by lineage and matrix, and how they wire into PI3K-AKT, MAPK, STAT3, or mTORC1 during real therapy exposure. Clinically, future directions of MDK should move from a single analyte to a compact prediction panel that combines plasma levels, tumor expression, and spatial features of myeloid and fibroblast cells to estimate response to PD-1 combinations, anti-angiogenic regimens, or platinum chemotherapy. Done well, this program can turn MDK from a descriptive hallmark of aggressive disease into a practical handle for selecting patients, strengthening combinations, and converting resistant disease into controllable disease.

## Acknowledgments

This study was supported by an 10.13039/100000054NCI grant to Vaishali Kapoor (K22CA234404).

## Author contributions

All authors wrote and revised this review article.

## Declaration of interests

The authors declare no competing interests.
